# The Roles of Gene Duplication, Gene Conversion and Positive Selection in Rodent *Esp* and *Mup* Pheromone Gene Families with Comparison to the *Abp* Family

**DOI:** 10.1371/journal.pone.0047697

**Published:** 2012-10-19

**Authors:** Robert C. Karn, Christina M. Laukaitis

**Affiliations:** Department of Medicine, College of Medicine, University of Arizona, Tucson, Arizona, United States of America; National Institute of Allergy and Infectious Diseases, United States of America

## Abstract

Three proteinaceous pheromone families, the androgen-binding proteins (ABPs), the exocrine-gland secreting peptides (ESPs) and the major urinary proteins (MUPs) are encoded by large gene families in the genomes of *Mus musculus* and *Rattus norvegicus*. We studied the evolutionary histories of the *Mup* and *Esp* genes and compared them with what is known about the *Abp* genes. Apparently gene conversion has played little if any role in the expansion of the mouse Class A and Class B *Mup* genes and pseudogenes, and the rat *Mup*s. By contrast, we found evidence of extensive gene conversion in many *Esp* genes although not in all of them. Our studies of selection identified at least two amino acid sites in β-sheets as having evolved under positive selection in the mouse Class A and Class B MUPs and in rat MUPs. We show that selection may have acted on the ESPs by determining *K_a_*/*K_s_* for Exon 3 sequences with and without the converted sequence segment. While it appears that purifying selection acted on the ESP signal peptides, the secreted portions of the ESPs probably have undergone much more rapid evolution. When the inner gene converted fragment sequences were removed, eleven *Esp* paralogs were present in two or more pairs with *K_a_*/*K_s_* >1.0 and thus we propose that positive selection is detectable by this means in at least some mouse *Esp* paralogs. We compare and contrast the evolutionary histories of all three mouse pheromone gene families in light of their proposed functions in mouse communication.

## Introduction

The availability of an increasing number of mammalian genome sequences has greatly enhanced our ability to investigate evolutionary processes and thereby advanced our understanding of gene evolution. Those genes not preserved as single copies in both primate and rodent lineages are subject to frequent duplication, deletion and pseudogene formation [1]–[3]. Conserved genes are likely to possess functions that are shared by primates, rodents, and, in all likelihood, by most mammals. By contrast, frequently duplicated genes are more often associated with adaptation and functional innovation [1], [4], [5]. They often show the footprints of positive selection in elevated ratios of nonsynonymous to synonymous nucleotide substitutions (*d_N_* /*d_S_*; sometimes reported as the rate *K_a_*/*K_s_*; [6]) in their coding regions [7]–[11]. Gene deletion and pseudogene formation events are rare, except among genes that have also been subject to duplication [2], [3], [12]. When these events are present, the affected gene region may show copy number variation and more volatility than other gene regions of similar size [13]. Prevalent among rapidly evolving genes are those involved in immunity, reproduction, chemosensation and toxin metabolism [1].

A great deal of interest has been focused on reproductive proteins encoded by genes, sometimes called speciation genes, that are associated with signatures of positive selection [14]–[17] and that have functions thought to promote reproductive isolation among closely related species [18], [19]. Special emphasis has been given to reproductive genes involved in postzygotic isolation but relatively little to those involved in prezygotic isolation, e.g. proteins with functions such as mediating mate choice [20], [21]. And yet there are examples of gene duplication acting as a major source of new gene functions involved in mate selection at the individual and population levels. Among these are three rodent pheromone protein families encoded by genes that have undergone extensive gene duplication in mice, rats and perhaps other members of Glires (see for example [22]). Some of the proteins encoded by all three gene families affect mate selection in one way or another, thus directly impacting gene exchange and thereby evolution and potentially speciation. These three gene families encode the androgen-binding proteins (ABPs), the exocrine-gland secreting peptides (ESPs) and the major urinary proteins (MUPs). Recently, mammalian communication by pheromones has received much attention that has been focused on mechanisms of communication and the behavioral responses they elicit in the house mouse, *Mus musculus* and other rodents. For this reason, we compared the evolutionary trajectories of three house mouse gene families that have been implicated in the production of proteins with pheromonal functions. Most studies to date have focused on defining the function of the members of one of these three families with little or no consideration of the roles played by the other two. It is our hope that comparing and contrasting the evolutionary histories of these three families may lead to a better understanding of the relative contribution of each to mouse behavior, particularly behavior involving mating and thereby directly influencing the animal’s contribution to the gene pool. Because all three mouse gene families have counterparts in the rat genome, the rat genes were included in this study where possible.

ABPs have been shown to mediate assortative mate selection, based on subspecies recognition that potentially limits gene exchange between subspecies where they meet ([21], [23]; reviewed in [24]) and there is evidence that ABP constitutes a system of incipient reinforcement where subspecies make secondary contact, the house mouse hybrid zone in Europe [25]. ESPs are small rodent proteinaceous pheromones [26]. Female mice respond to direct facial exposure to an ESP, expressed in male exorbital lacrimal glands and released into tear fluid, by up-regulating *c-Fos* and *egr1* gene expression in vomeronasal sensory neurons [27]. There is now evidence that mouse ESP1 enhances female sexual receptive behavior, lordosis (the position that some female mammals display when they are ready to mate), upon male mounting and copulation [28]. The MUPs are a family of lipocalins shown to mediate female recognition of potential mates (for a review, see [29]). Each adult mouse expresses a pattern of 8–14 different MUP isoforms in its urine, which is determined by its genotype and by its sex because some *Mup* genes show sex-limited expression [29]. This individual recognition profile has been likened to a protein “bar code” [30]–[34]. MUPs have been implicated in male–male aggression [35], [36] and other studies have shown that both MUPs [37], and a hypothetical MUP peptide formed from the six N-terminal residues EEARSM [38], [39], are androgen-regulated nonvolatile compounds capable of accelerating puberty in female mice.

The ABP, MUP and ESP pheromones have different molecular properties. The ABPs are dimers composed of an alpha subunit disulfide-bridged to a beta/gamma subunit [40], [41]; (see [22] for nomenclature) unlike the MUPs and ESPs, which are single peptide chains. The ABP subunits are four-helix bundles that take the boomerang form typical of the secretoglobin superfamily [42], while the MUPs are lipocalins with the dominant β-sheet secondary structure folded into β-barrels [29], [33], [43]. Both bind small ligands, ABP in the cleft formed by the association of the two subunits [42], [44] and MUP in the internal β-barrel [29], [33], [43]. While there has been no study of the conformation of the secreted ESP peptides, their small size and highly diverged sequences make it likely that they are random coils following secretion. The same arguments suggest that they probably do not bind ligands as do the MUPs and ABPs.

What was previously known about the expansions of each of these three gene families in rodent and other genomes? The *Abp* gene arrangement is most often found as an <alpha-beta/gamma> pair (<*Abpa*-*Abpbg*> abbreviated <*a*-*bg*> with arrows pointing in the 3′ directions; [22], [45]). The basal situation in the mammal genome appears to be a single such pair, sometimes with one or more pseudogenes, for example in the little brown bat, horse, cat, dog, squirrel and tree shrew, although independent expansions involving multiple alpha and/or beta/gamma paralogs have been observed in opossum, cattle, mouse, rat and rabbit [22]. The primate lineage, including human, chimpanzee, and possibly macaque, apparently has only a pseudogenized pair [22]. A single *Mup* gene without evidence of a pseudogene(s) appears to be the basal situation in mammals such as the dog, pig, baboon, chimpanzee, bush-baby and orangutan but not in humans where only a pseudogene with an altered donor splice site has been observed [33]. However, at least two lineage-specific expansions have been found, one in the horse (three *Mup* paralogs) and the other in the grey mouse lemur (*Microcebus murinus*; at least two *Mup* gene paralogs and one presumptive pseudogene; [33]). In the case of *Esp* genes, only the mouse, rat and human genomes have been interrogated with the finding of 38, 10 and 0 paralogs, respectively [27], so it is not possible to determine the basal condition in mammals more widely. The information that existed prior to this study suggested that the gene expansions of the *Abp*s [22], [45] and the *Mup*s [33] happened independently in *M. musculus* and *R. norvegicus*. This contrasts with the suggestion that *Esp* gene expansion, at least for many/most paralogs, began in an ancestor predating the *Mus*/*Rattus* divergence [27]. In any event, the one characteristic shared by all three gene families is that they have greatly expanded in mouse and to a lesser extent in rat.

In a previous report, we described the evolutionary history of the *Abp* gene family, observing copy number variation among the most recently duplicated *Abp* genes and suggesting that there is substantial volatility in this gene region [13]. We concluded that groups of these genes behave as low copy repeats (LCRs), duplicating as relatively large blocks of genes by nonallelic homologous recombination (NAHR). Our analysis of gene conversion suggested that it did not contribute to the very low or absent divergence among the paralogs duplicated in this way. Others have studied aspects of the evolutionary histories of the *Mup* [33], [43] and *Esp* [27] genes. Two groups studying the *Mup* genes speculated that gene conversion played an important role during the duplication of the closely related members of the Class B *Mup* genes ([33], [43]; we use here the nomenclature of [33]). In addition to envisioning a role for gene conversion in *Mup* gene evolution, Mudge *et al* [43] speculated that NAHR might also have played a part. Studies of the *Esp* gene family are much more recent and until now no detailed study of their evolutionary history was available. We report here the first attempt to assess the contributions of gene conversion and selection to the evolutionary history of this family of pheromone genes. In addition, we revisited the question of the mechanisms behind the evolutionary histories of the *Mup*s and compare our findings with what is known about the evolutionary history of the *Abp* gene family and what we have learned about the *Esp*s.

We focused our study on applying tests for gene conversion and for the role of selection on these extensively expanded gene families. We present new findings, some of which disagree with speculation presented by others, and we compare and contrast the evolutionary histories of all three mouse pheromone gene families in light of their proposed functions in mouse communication.

## Materials and Methods

### Accession of MUP and ESP Sequence Data

Mouse MUP protein sequences were accessed with their TPA numbers and their gene sequences were obtained from the associated links. *Mup* gene coordinates were found by using their gene sequences as search strings in the BLAT tool of the UCSC genome browser [46] and are shown in **Table S1**. The mRNAs corresponding to each *Mup* gene were found by submitting their protein sequences to tBLASTn and/or by reconstructing them from translations of exons in their genes. Mouse and rat ESP amino acid and nucleotide sequences were obtained from NCBI using the accession numbers reported in [27]. *Esp* mRNA accession numbers were used to obtain their mRNA nucleotide sequences which were in turn used as search strings in the BLAT tool of the UCSC genome browser [46] to obtain their gene coordinates and sequences. These and their protein accession numbers are shown in **Table S2**.

### Completion of Rat ESP Amino Acid Sequences – Finding Signal Peptides

Previously published rat ESP amino acid sequences included the Exon 3 coding region only [27]. In order to complete the amino acid sequences and the Exon 2 coding region, we searched upstream DNA sequences using the following strategy: 1) the published accession number was used to obtain the DNA sequence (i.e. third exon DNA) of a rat ESP; 2) the “Get DNA” function was used to add more DNA sequence (beginning with 6 kb) to the upstream end of the DNA and this was downloaded to DNAsis Max for processing; 3) the entire downloaded DNA was translated into all three frames and the sequences were individually searched for the EG (GluGly), EE (GluGlu) or DG (AspGly) pair that occurs at or before positions 21 and 22 in the signal peptide; 4) sequences were retained only if they began with Met, ended with a GT pair after the EG, EE or DG pair and otherwise contained only amino acids with hydrophobic side chains, as well as Ser and/or Thr. No such candidate sequence was found for *rEsp2*, probably because there is a 2.36 kb gap in the rat genome sequence 4.38 kb upstream of Exon 3, where most of the candidates were found in other *rEsp* genes. Likewise, we could not find a candidate signal peptide for *rEsp10*, even scanning 20 kb proximal to Exon 3. In this case, however, there was no gap and we can only conclude that it was obliterated subsequent to duplication.

### Determination of Signal Peptide Cleavage

Signal peptide coding sequences were removed from mRNA coding sequences for the purpose of evaluating the role of selection on the secreted versions of the MUP and ESP proteins. We used three signal peptide prediction algorithms: SignalP 4.0, www.cbs.dtu.dk/services/SignalP/; [47]; Sig-Pred, bmbpcu36.leeds.ac.uk/prot_analysis/Signal.html [48] and Signal 3L, www.csbio.sjtu.edu.cn/bioinf/Signal-3L/; [49].

### Detecting Gene Conversion

**Figure 1:**
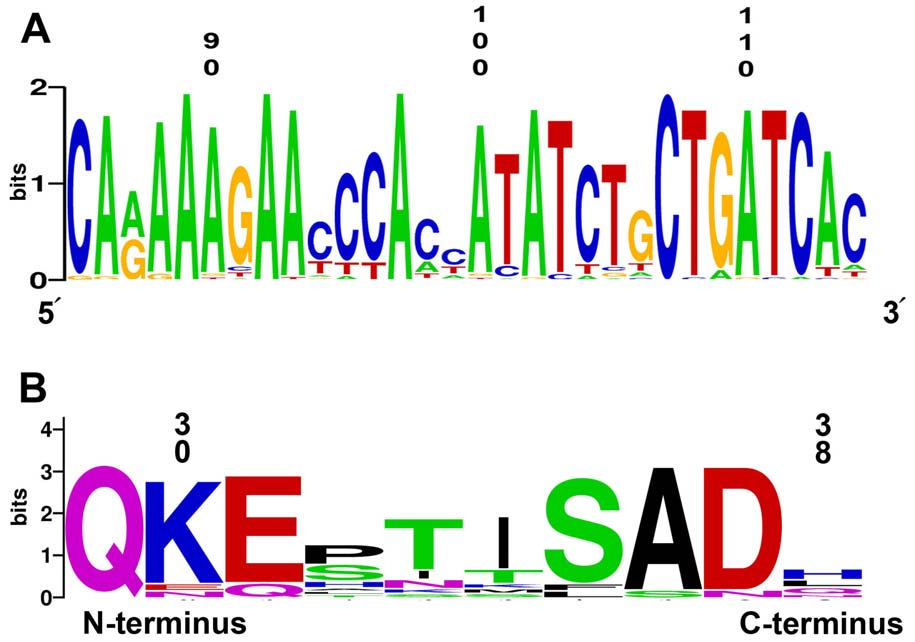
WEBLOGO of the inner fragment shared by 21/38 mouse and 9/10 rat *Esp* genes. Panel A: The nucleotide sequence in the gene-converted region for the expressed mouse *Esp* genes and the rat *Esp* genes involved in gene conversion. Panel B: The translation of the inner fragment sequence. The y-axis values are bits, the maximum entropy for the given sequence type (log_2_ 4 = 2 bits for DNA/RNA, log_2_ 20 = 4.3 bits for protein; weblogo.berkeley.edu/info.html).

The program GENECONV (www.math.wustl.edu/sawyer/geneconv/gconvdoc.pdf; [50]) provides a means of determining the extent of gene conversion in a set of sequences by seeking aligned DNA or protein segments for which a pair of sequences is sufficiently similar to suggest that gene conversion occurred. These are classified as inner or outer fragments. Inner fragments are evidence of a possible gene conversion event between ancestors of two sequences in the alignment. Outer fragments are runs of unique sites that may be evidence of past gene conversion events that originated from outside of the alignment or else from within the alignment but such that evidence of the source has been destroyed. GENECONV designates the location(s) of the region of sequence affected and gives the user the option to introduce one or more mismatches by setting the *gscale* from the default value of 0 (none allowed) to 1 or more. This potentially extends the sequence in question and may also increase the number of fragments observed, but the user must beware that more noise may also be introduced into the result. Another important caveat for using GENECONV is that it does not perform well when the paralogous sequences are nearly identical, whether that is due to extensive gene conversion or simply tandem duplication so recent that there has been little divergence between the paralog products. We aligned sequences with CLUSTALX [51], [52] and used GENECONV to search for gene conversion tracks. GC content of the mouse and rat gene regions was determined using an online calculator provided by EnCore Biotechnology, Inc. (www.encorbio.com/protocols/Nuc-MW.htm). The representation of the inner fragment in **Fig. 1** was produced with Weblogo 3 (weblogo.threeplusone.com/).

### Data Analysis

The sequences encoding the mature peptides following the signal-sequence cleavage sites were aligned using CLUSTALX and, separately, CLUSTALW in DNAsis Max 2.0. Phylogenetic trees were constructed from the alignments with PAUP* [53] using neighbor-joining (NJ) distance parameters with Jukes-Cantor correction and these were displayed in TreeView [54]. Nucleotide divergences were calculated using Mega 5.05 [55] with the Kimura correction for multiple hits and a transition:transversion ratio of 2. The distances and their standard errors were compared by a modification of a one-tailed t-test with infinite degrees of freedom [56].

**Figure 2:**
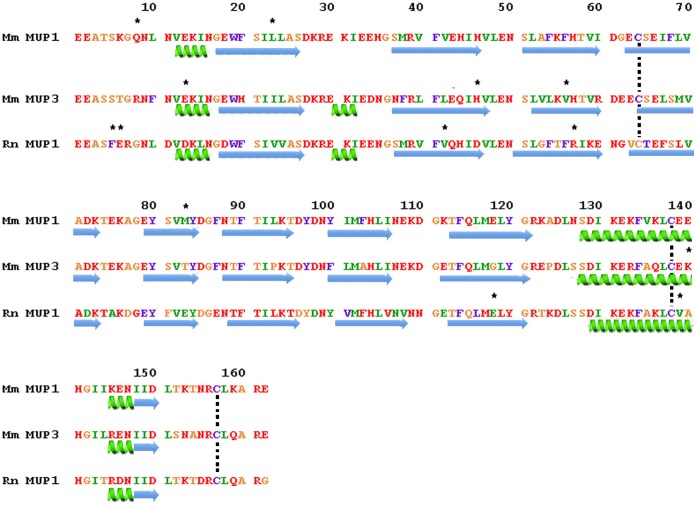
CODEML analysis of rodent MUPs showing a comparison of the ω+ sites. The mouse Class A, Class B and rat MUPs were analyzed independently and mapped on the mouse MUP1 and MUP3 and rat MUP1 sequences, respectively. The numbering system begins with the first amino acid residue of the cleaved, secreted protein. Arrows below the sequences denote β-sheet secondary structure and coils denote alpha helix. Asterisks mark the sites with posterior probabilities greater than 0.9. Vertical dashed lines show the conserved ½ Cys residues among the three sequences. The amino acid color coding is to facilitate comparison of amino acid residues at specific sites from sequence-to-sequence.

**Figure 3:**
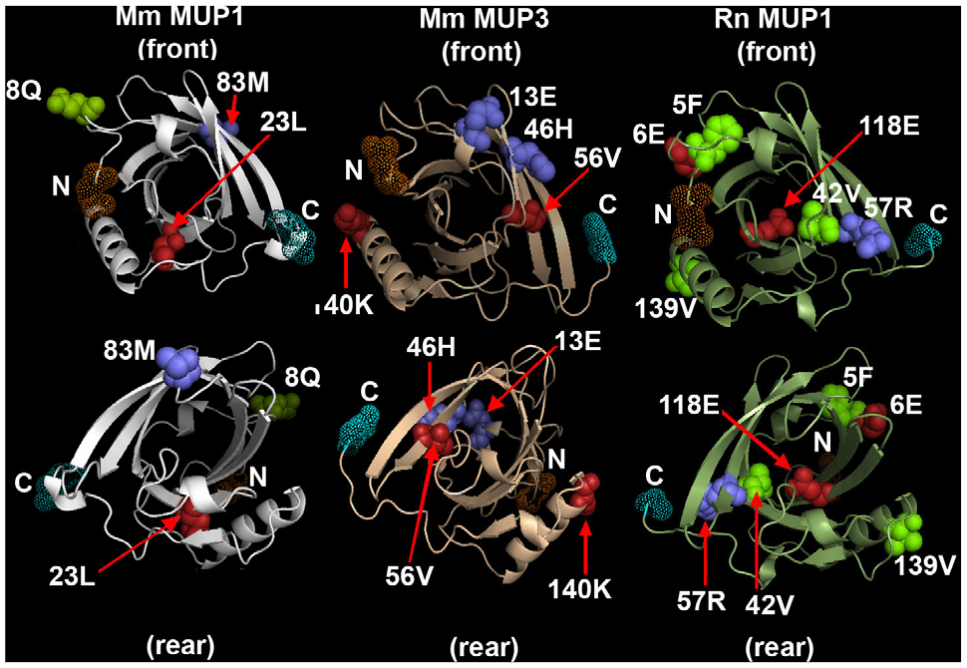
Positive selection on rodent MUPs. Selected sites are plotted on molecular models of mouse MUP1 (left), MUP3 (center) and rat MUP1 (right), representing the mouse Class A, Class B and rat MUPs. Both mouse MUP1 and MUP3 were mapped on the d1znda1 model and rat MUP1was mapped on the d2a2ua model with PyMol. Table 4 lists the probability of selection on specific residues. Residues with a BEB posterior probability >99% are in red; a BEB posterior probability >95% are in green; and a BEB posterior probability >90% are in blue. In all the models, α-helices are shown as spiral tapes and β-sheets are shown as flat arrows. The eight-sheet β-barrel can be seen in the center of each model. At least two of the selected sites map to different β-sheets in the β-barrel of all three structures.

Positive selection was assessed in the program CODEML in the PAML 3.14 package [57], [58]. The phylogeny of Chevret *et al* [59] was used for the mouse species for initial PAML tests and the three subspecies of *M. musculus* were treated as an unresolved polytomy. For each gene, three different comparisons of neutral and selection models gave similar results (M1 vs. M2, M7 vs. M8, and M8A vs. M8 [11], [60], [61]). Model M1 (neutral) allows two classes of codons, one with *d_N_*/*d_S_* over the interval (0,1) and the other with a *d_N_*/*d_S_* value of one. Model M2 (selection) is similar to M1 except that it allows an additional class of codons with a freely estimated *d_N_*/*d_S_* value. Model M7 (neutral) estimates *d_N_*/*d_S_* with a beta-distribution over the interval (0,1), whereas model M8 (selection) adds parameters to M7 for an additional class of codons with a freely estimated *d_N_*/*d_S_* value. M8A (neutral) is a special case of M8 that fixes the additional codon class at a *d_N_*/*d_S_* value of one. The three-dimensional structures of mouse MUP1 and MUP3 and rat MUP1 were modeled using the PHYRE 2.0 threading program (www.sbg.bio.ic.ac.uk/phyre/; [62]) and the display was modified to produce **Fig. 2**. The resulting models were visualized and sites under positive selection were mapped to the structural models in **Fig. 3** using PYMOL (www.pymolorg/; open-source 1.2.8).

We conducted a *K_a_*/*K_s_* analysis of *Esp* GENECONV fragments involving *Esp24* by first sorting the 25 fragments involving *Esp24* Exon 3 and retaining only those putatively expressed [27]. We translated all the Exon 3 sequences and removed the first stop codon and the sequence downstream of that. The sequences were aligned and the 30 bp inner fragment identified by GENECONV, *gscale = *1 removed from each, after which they were realigned with their starting sequence to ensure that their alignments matched in all regions. Finally, we exported the aligned sequences with and without the gene-converted fragment to a FASTA file and ran DNAsp (http://www.ub.edu/dnasp/ [63]) to obtain their pairwise *K_a_*/*K_s_* values.

## Results

### The N-termini of Mouse and Rat Mup and Esp Genes

Before we could undertake evolutionary studies of the mouse and rat *Mup* and *Esp* genes, it was necessary to ascertain the N-terminus of each of the secreted proteins they encode because selection, gene conversion and other evolutionary mechanisms may operate differently on the cleaved, secreted protein than on the signal peptide [64]. In the case of the MUPs, the work of others has shown that cleavage C-terminal to the first Ala residue results in a consistent GluGlu doublet starting the secreted protein sequence [33]. We used this as the signal peptide cleavage point for mouse and rat MUPs.

Determining the starting residue of the ESP secreted protein was more difficult because the cleaved ESP peptide resulting from secretion has not been reported although it was suggested that the entire coding region beyond the signal peptide is found in the third exon [27]. That predicts that cleavage of the signal peptide should occur C-terminal to the last residue encoded by Exon 2, an Arg residue. This seems unlikely because most signal peptide sequences are cleaved C-terminal to a residue with a simple side chain, e.g. Ala, and not a complex side chain, such as that found in Arg. To better predict the cleavage site of the signal peptide, we employed three different algorithms for detecting the point of its removal from the protein sequences (see Methods) and we worked with the fifteen mouse *Esp* genes (*Esp*s *1*, *3*, *4*, *5*, *6*, *8*, *15*, *16*, *18*, *23*, *24*, *31*, *34*, *36*, *38*) reported as expressed [27]. The three algorithms predicted cleavage of the signal peptide on the C-terminal side of either the Thr or the Gly residue that occurs just before the Arg residue encoded at the end of Exon 2. Gly is the consensus residue in these sequences and so, for the purposes of this study, we removed the signal peptide C-terminal to that. Thus we assigned the last residue encoded in Exon 2, usually but not always an Arg, as the first residue of the secreted protein.

### Ascertainment of Intron b Sequences of Rat Esp Genes

The rat *Esp* gene sequences that appear in GenBank lack signal peptides [27]. In order to compare the gene conversion results for *Esp* Exon 3 analysis to a noncoding part of the gene, it was necessary to determine the starting and ending points of the intron (intron b) lying between Exons 2 and 3. We took advantage of the relatively well-conserved mouse ESP signal peptide amino acid sequences to devise a method for finding probable signal peptide coding sequences for rat *Esp* genes (see Methods) and were able to find putative signal peptides for rat ESPs 1, 3, 4, 5, 6, 7, 8 and 9 but not for 2 or 10. As in the case of the second exons of the mouse *Esp* genes, these sequences have GluGly or GluGlu pairs at the end of the exons and we predict that the Gly or the second Glu is the consensus residue for signal peptide removal. However, unlike the mouse gene sequences, only the first base of the codon for the first residue of the secreted protein appears before the GT donor splice site and the remaining two bases begin the third exon sequences. We pieced together putative mRNA sequences using these new second exons and the third exons in GenBank, and translated them to obtain the putative amino acid sequences of the translated proteins before they are secreted (**Dataset S1**). These mRNA sequences were used with BLAT to obtain the intron b sequences lying between the two exons.

### Evidence is Sparse for Gene Conversion in Mouse and Rat Mup Genes

We began our study of the evolutionary history of the mouse and rat *Mup* gene families by asking if gene conversion has contributed significantly to sequence identity in either of them as has been previously proposed for the Class B *Mup*s [33], [43]. Gene conversion in *Mup*s was first proposed by Clark *et al* [65] before algorithms, such as GENECONV were available to detect it. As useful a tool as the program is, however, it has been shown that GENECONV has poor power to detect conversion events when divergence between duplicates is very low [66], whether that is due to extensive gene conversion or simply tandem duplication so recent that there has been little divergence between the paralog products. GENECONV has also been shown to have high false negative rates [67].

We adopted the *Mup* gene and MUP protein nomenclature of Logan *et al* [33] because we obtained their sequences from NCBI. The gene coordinates are listed in **Table S1**. The results of our GENECONV analysis of mouse Class A, Class B, Class B pseudogenes and rat *Mup* paralogs are shown in **Table 1**. In summary, we found few inner fragments (conversion between genes within the alignment) and even fewer globally significant outer fragments (conversion with genes outside the alignment).

**Table 1:** GENECONV results for *Mup* paralogs.

Gscale = 0						
*Mup* Paralogs	Inner fragments		Paralogs involved	Outer fragments	Paralogs involved	
Mouse Class A	1		*Mup25*/*Mup26*	0		
Mouse Class B	1		*Mup12*/*Mup8*	0		
Mouse Class B pseudogenes	1		*Mup9ps*/*Mup4ps*	1	*Mup14ps*	
Rat	2		*Mup13*/*Mup4* & *Mup10*/*Mup4*	1	*Mup1*	
**Gscale = 1**						
***Mup*** ** Paralogs**	**Inner fragments**	**Paralogs involved**		**Fragment position** a	**Outer fragments**	**Paralogs involved**
Mouse Class A	4	*Mup1*/*Mup18*		369–613	0	N/A
		*Mup1*/*Mup25*		2194–2296		
		*Mup25/Mup26*		1725–2142		
		*Mup2/Mup24*		16–483		
Mouse Class B	2	*Mup9/Mup5*		1544–2487	0	N/A
		*Mup12*/*Mup8*		1857–2812		
Mouse Class B pseudogenes	1	*Mup4/Mup2*			0	N/A
Rat	1	*Mup4/Mup2*			0	N/A

a Only for number of fragments >1.

The first set of GENECONV analyses (above) allowed no mismatches. The results we obtained when we reran the GENECONV analysis allowing a single mismatch (*gscale* = 1) are also shown in **Table 1**. The mouse Class A *Mup*s experienced a four-fold increase in their inner fragments, however, there was minimal overlap in only two of the converted regions of the alignment for the four fragments (**Table 1**). We conclude that gene conversion made a minimal, but not nonexistent, contribution to the evolution of the murid rodent *Mup* genes and found little support for the idea that gene conversion significantly shaped the mouse Class B *Mup*s [33], [43].

We calculated the GC content of the mouse and rat *Mup* gene regions because sequences undergoing frequent gene conversion, either ectopic or allelic, are expected to become GC rich [68], [69]. We found the following average GC contents in the four sets of *Mup* paralogs: Class A Mup genes, 39.89%; Class B genes, 41.31%; Class B pseudogenes, 39.76%; and rat genes, 45.46%. These GC contents in the various rodent *Mup* gene regions are relatively low compared with genes undergoing gene conversion [68], [70], [71], although there is conflicting data on whether increased GC content is consistent with gene conversion [66]. Nonetheless, we feel that the low GC contents support the conclusion from the GENECONV analyses of the whole *Mup* genes that conversion has contributed minimally to the expansions of these gene families.

### There is Substantial Evidence of Gene Conversion in Mouse and Rat Esp Genes

By contrast with our *Mup* gene findings, we observed significant evidence of gene conversion between mouse and rat *Esp* genes. The mouse and rat *Esp* gene sequences that we used in our study were those deposited in NCBI by Kimoto *et al* [27] and their gene coordinates are listed in **Table S1**. We tested *Esp* Exon 3, which encodes nearly the entire secreted peptide in 38 mouse and 10 rat *Esp* genes. We pooled Exons 3 of mouse and rat *Esp* genes for this purpose because phylogenetic evidence has been produced for the divergence of many/most *Esp* paralogs in the murid rodent lineage before the divergence of *M. musculus* and *R. norvegicus* [27].

When we used the default *gscale* setting of 0 for mismatches to analyze the 48 rodent *Esp* genes, the GENECONV program predicted fifteen inner and no outer globally significant fragments (**Table 2**). The fifteen inner fragments did not involve random pairing of the 48 paralogs tested. Rather, mouse *Esp24* was a member of eight pairs (53% of the total; **Table 2**) while the other pairs involved three or fewer of the same paralog. Of the fifteen inner fragments, seven involved two mouse paralogs, five involved a mouse and a rat paralog and three involved two rat paralogs. The five mixed-species fragments support the conclusion that divergence of many/most *Esp* paralogs in the murid rodent lineage occurred before the divergence of *M. musculus* and *R. norvegicus* [27].

**Table 2:** Mouse and rat *Esp* fragments identified by GENECONV.

	*gscale* setting	Number of inner fragments	Number of outer fragments	Mean length^a^	Median length^a^	Number of inner fragments involving Esp24
Exon 3	0	15	0	18.8	18	8
	1	70	0	31.3	30	25
intron b	0	12	0	27.2	16	1
	1	18	0	825.8	144	2

a Mean and median lengths shown in Dataset S2.

These fifteen inner fragments obtained with the default settings consistently identified a sequence that spans ∼20 bp of the first 128 bp found in the third exon of all 48 rodent *Esp*s. GENECONV analysis with *gscale* = 1 revealed 70 globally significant inner fragments and no outer fragments, a 4.7-fold increase in inner fragments (**Table 2**). The single mismatch allowed an additional ∼10 bp proximal to the original ∼20 bp for a fragment of total length ∼30 bp. Twenty five of the 70 inner fragments (36%) involved mouse *Esp24* while 17 involved rat *rEsp9* (24%); these two categories account for 42 of the 70 inner fragments (60%). The remaining 28 inner fragments were distributed among eight other groups with involvement of from 1–6 other *Esp* genes. This strong bias in *Esp* paralog associations in inner fragments (Chi square; P<0.0001), the primary association being with *Esp24*, suggests that there may have been a selective advantage in gene conversion of ∼30 bp (**Dataset S2**) of one or more paralogs during the extensive expansion of the rodent *Esp* gene family.

Finally, we reran the GENECONV analysis with *gscale = *2, which returned only 29 globally significant inner fragments and no outer fragments (not shown), a decrease over the *gscale = *1 result. Thus the greatest number of fragments (all inner fragments) was returned with a *gscale* setting of 1 and over all the *Esp* genes, 21/38 mouse and 9/10 rat *Esp* genes were involved in inner fragments while 17/38 mouse and 1/10 rat *Esp* genes were not. We suggest that these data provide substantial evidence for gene conversion among more than half the mouse 38 *Esp* genes and nearly all ten of the rat genes.

Allowing the single mismatch by changing the *gscale* setting from 0 to 1 also increased the gene-converted sequence span by 50% (∼10 bp) on the 5′ side of the original span. In fact, the most intriguing finding of this analysis is that all 70 inner fragments identified the same ∼30 bp sequence in the alignment within several bases in either direction (5′ or 3′). **Figure 1A** shows the consensus nucleotide sequence in the gene converted region for the expressed mouse *Esp* genes and the rat *Esp* genes involved in gene conversion. The consensus translation of that sequence is shown in **Fig. 1B**. It appears that the nucleotide sequence nearest the flanks of the converted region is the most conserved, which might be expected if it is responsible for the alignment leading to the conversion process.

We also performed GENECONV analysis of intron b, which connects Exon 2 (mostly signal peptide) with Exon 3 (most of the secreted ESP peptide). We were not able to include all possible alignments, in part because we were not able to find Exon 2 of two rat *Esp* paralogs (see above) and therefore could not identify the GT donor splice site of the introns, and in part because ClustalX was unable to align *rEsp4* and *rEsp8* introns b with the others. With the default *gscale* setting of 0, GENECONV found twelve inner and no outer fragments in the intron b analysis (**Table 2**) but there were far fewer fragments involving the same partner compared to the Exon 3 coding region analysis above (e.g. only one involving *Esp24*; **Table 2**). With a *gscale* setting of 1, GENECONV found 18 inner and no outer fragments in the intron b analysis, a 1.5-fold increase in inner fragments. Again the number of fragment pairs involving the same paralog partner was low (e.g. only two involving *Esp24*; **Table 2**). Moreover, groups of sequences identified in the different fragments did not overlap in many cases, unlike the clearly identified consensus sequence found in our analysis of Exon 3. The widely different mean and median fragment lengths found in intron b reflect this lack of uniformity (**Dataset S3**).

In the case of the *Esp* genes, we observed that the GC content of paralogs involved in inner fragments found with the default *gscale* differed significantly from the GC content of those that were not involved (one-tailed t test, P = 0.02 for both 15 inner fragments and 70 inner fragments; **Table 3**). These GC values are not particularly high compared to other genes that have undergone gene conversion but the significantly higher GC content of those involved in both the fifteen inner fragments (39%) obtained with *gscale = *0 and the 70 (37%) obtained with *gscale = *1supports the GENECONV evidence for gene conversion among some *Esp* genes. The same was not true of the GC content of those involved in the intron b analysis. There the GC contents of the twelve inner fragments did not differ significantly from that of those not involved in gene conversion (36% and 34%; one-tailed test, P = 0.12; **Table 3**).

**Table 3:** GC content of mouse and rat *Esp* genes.

	*gscale* setting	Innerfragments	Mean GC content ofparalogs involved	S.D.	Mean GC content ofparalogs not involved	S.D.	One-tailed test
Exon 3	0	15	0.39	0.062	0.35	0.029	P = 0.02
	1	70	0.37	0.05	0.35	0.03	P = 0.02
intron b	0	12	0.36	0.041	0.34	0.045	P = 0.12

### The Role of Selection in Rodent Mup Gene Evolution

We considered the possibility that the two subfamilies of *M. musculus Mup* genes evolved under different selection regimens and we began by comparing nucleotide divergence of the exons to that of the introns. Both intron divergence and the synonymous nucleotide sites in the coding region (represented by *d_S_*) are for the most part thought to be free of selective constraints and thus their values should be similar. This is because comparisons of homologous DNA sequences for many different genes reported by Hayashida and Miyata [72] showed that silent positions of protein-encoding regions (estimated by *Ks* or, alternatively, *dS*) and introns (which we estimated with nucleotide divergences) evolve at high and remarkably similar rates for different genes. Those authors concluded that the evolutionary clocks at the DNA level in such divergent blocks as silent positions and introns run at essentially the same rates for many different genes over a long period of evolutionary time.

In the case of positive selection, by contrast, the coding region is predicted to show higher nucleotide variability than the introns. The prediction is the opposite in the case of purifying (negative) selection: the coding region should show reduced nucleotide variability compared to the introns. **Table 4** shows the results of these comparisons, wherein we removed the signal peptide coding region from consideration because it is expected to be under different selective constraints than the region encoding the secreted protein. As expected, the overall nucleotide divergence values that we calculated for the *M. musculus* Class A and B *Mup* concatenated introns agree well with the *d_S_* values of Logan *et al* [33]. Nucleotide divergences of the *M. musculus* Class A *Mup* exons and introns were not significantly different from each other (one-tailed t test modified from [56]; P > 0.25) and the nucleotide divergence value that we calculated for the Class A concatenated introns agrees well with the *d_S_* values of Logan *et al* [33]. In the case of the *M. musculus* Class B *Mup* genes, the exons show significantly less nucleotide divergence than the introns (one-tailed t test modified as before; P = 0.005). As in the case of the *M. musculus* Class A *Mup*s, the nucleotide divergence values that we calculated for the *M. musculus* Class B concatenated introns agree well with the *d_S_* values of Logan *et al* [33]. **Table 4** also contains an analysis of *R. norvegicus Mup* exons and introns. The nucleotide divergence in the rat exons significantly exceeds that in the introns (one-tailed t test as before; P < 0.001). In this case, however, the divergence of the concatenated introns is less (0.059) than the *d_S_* value (0.098) of Logan *et al* [33]. In light of the lack of evidence for gene conversion, our data suggest that the exons of the mouse Class A and rat *Mup* genes have experienced significant nucleotide substitution in their evolutionary histories while, by comparison, the mouse Class B *Mup* genes seem to have been under purifying selection.

**Table 4:** Divergences in Exons and Introns of *Mup* genes.

*Mus musculus Mup* Class A divergences							
Exon 1 (coding only)	Exon 2	Exon 3	Exon 4	Exon 5	Exon 6		
0.162	0.172	0.257	0.09	0.117	0.103		
s.e. = 0.089	s.e. = 0.052	s.e. = 0.116	s.e. = 0.037	s.e. = 0.043	s.e. = 0.121		
	**intron a**	**intron b**	**intron c**	**intron d**	**intron e**	**Concatenated introns** a	***d_S_*** b
	0.162	0.121	0.175	0.123	0.109	0.143	0.133
	s.e. = 0.019	s.e. = 0.008	s.e. = 0.012	s.e. = 0.013	0.018	s.e. = 0.005	
***Mus musculus Mup*** ** Class B divergences**							
**Exon 1 (coding only)**	**Exon 2**	**Exon 3**	**Exon 4**	**Exon 5**	**Exon 6**		
0.019	0.015	0.006	0	0.013	0		
s.e. = 0.020	s.e. = 0.012	s.e. = 0.006	s.e. = 0	s.e. = 0.013	s.e. = 0		
	**intron a**	**intron b**	**intron c**	**intron d**	**intron e**	**Concatenated introns** a	***d_S_*** b
	0.016	0.011	0.021	0.025	0.005	0.017	0.018
	s.e. = 0.004	s.e. = 0.002	s.e. = 0.003	s.e. = 0.005	s.e. = 0.002	s.e. = 0.002	
***Rattus norvegicus Mup*** ** divergences**							
**Exon 1 (coding only)**	**Exon 2**	**Exon 3**	**Exon 4**	**Exon 5**	**Exon 6**		
0.056	0.064	0.136	0.078	0.032	0.074		
s.e. = 0.048	s.e. = 0.024	s.e. = 0.045	s.e. = 0.031	s.e. = 0.017	s.e. = 0.067		
	**intron a**	**intron b**	**intron c**	**intron d**	**intron e**	**Concatenated introns** a	***d_S_*** b
	0.051	0.062	0.062	0.052	0.07	0.059	0.098
	s.e. = 0.008	s.e. = 0.009	s.e. = 0.006	s.e. = 0.007	s.e. = 0.013	s.e. = 0.003	

a Using nucleotide differences of concatenated introns is more accurate than calculating an average over all introns.

b *d_S_* values taken from [33].

### Selection on MUP Amino Acid Sites

Logan *et al* [33] reported that mouse and rat *Mup* genes had pairwise *d_N_*/*d_S_* values consistent with a selective constraint acting on them, i.e. *d_N_*/*d_S_* less than 1.0 (*M. musculus* Class A *Mup*s, *d_N_*/*d_S_* = 0.769; *M. musculus* Class B *Mup*s *d_N_*/*d_S_* = 0.333 and rat *Mup*s *d_N_*/*d_S_* = 0.498). However, the averaging effect of *d_N_*/*d_S_* computed over all amino acid sites may result in a value less than 1.0 for a protein with a portion of sites under selection, so proteins with *d_N_*/*d_S_* values between 0.5 and 1.0 might still be evolving under positive selection. This is supported by experiments in which strong evidence of positive selection was revealed by a site-by-site test in proteins with overall *d_N_*/*d_S_* values that are elevated but less than 1.0 [73], [74].

To assess the role of positive selection in the three rodent *Mup* gene families, we employed the CODEML program from the PAML package ( [57], [75]; FASTA alignments are presented in **Dataset S4** and the gene trees are shown in **Fig. S1**). **Table 5** shows a summary of the CODEML results, which indicate that positive selection has acted at varying numbers of sites, designated ω+ sites, on the three sets of *Mup* paralogs. The ω + sites are mapped on sequences of the MUPs in mouse and rat shown in **Fig. 2**. At first glance, the locations where positively selected sites map in these three rodent MUP groups appear to have limited similarity, however, closer inspection reveals that there are at least two sites in each MUP group that map on β-sheet secondary structure in the β-barrel. To determine whether the positively-selected sites in the MUP sequences of the two mouse MUP subfamilies correspond to similar domains in their three-dimensional structures, we modeled them using the PHYRE threading program (**Table 6**) and visualized the resulting models with PYMOL (**Fig. 3**). The models show that each lipocalin has two or three selected sites on β-sheets in the barrel in the interior of the molecule (23L and 83M in mouse Class A; 56V and 46H in mouse Class B and 42V, 118E and 57R in the rat MUPs). The other selected sites were either near the N-terminus (8Q in mouse Class A; 13E in mouse Class B; 5F and 6E in rat) or the C-terminus (140K in mouse Class B; 139V in rat) and all of these were at least partially exposed on the surface of the protein. The overall conclusion from comparing the mouse and rat models is that at least two of the sites under positive selection in each map in the β-barrel where they could possibly influence the nature of the ligand preferentially bound. The other sites mapped on the surface, however, they were not all on the same face of the protein.

**Table 5:** Selection Test on *Mup* genes.

Gene	Ratio of *d_N_*/*d_S_* (%Codons)^a^	*P* Value All Genes^b^	Codon Sites Under Selection
*Mm MUP* Class A genes	3.6 (24.3%)	0.0029	**8Q**, **23L**, 83M
*Mm MUP* Class B genes	8.2 (6.7%)	<0.0003	13E, 46H, **56V**, **140K**
*Rn MUP* genes	4.3 (10%)	<0.0000	**5F**, **6E**, **24V**, 42V, **57R**, 60E, **118E**, 139V, **151L**

a The *d_N_*/*d_S_* ratio of the class of codons under positive selection is given with the percentage of codon sites predicted to be in that class.

b The P-value rejecting the model of neutral evolution (M8A) over that of selection (M8) is given.

c Sites with posterior probabilities greater than 0.9 are indicated in regular typeface; P > 0.95 indicated in bold typeface and P > 0.99 indicated in bold, underlined typeface.

**Table 6:** Mouse Genes Used to Produce Molecular Models.

Rodent gene	Accession Number	Chromosomal Location^c^ (strand)	Threaded Structure^a^ (results)^b^
*Mus musculus Mup1*	BK006638	chr4∶59957865–59960599 (−)	d1znda1 (100%; 157; 75%)
*Mus musculus Mup3*	BK006640	chr4∶60067530–60070300 (−)	d1znda1 (100%; 157; 98%)
*Rattus norvegicus Mup1*	NM_147215	chr5∶77660968–77663234 (−)	d2a2ua (100%; 158; 87%)

a The secreted sequences (i.e., signal sequences removed) were threaded for this study.

b Data consist of structural model, % confidence, length, and % identity.

c GRC38 coordinates.

### What are the Indications that Selection has been Involved in Rodent Esp Gene Evolution?

Mouse and rat *Esp* genes differ in many ways from the *Mup* genes of the two species. The *Esp* genes are much smaller than *Mup* genes and vary widely from each other in the lengths of the secreted ESP peptides they encode. Although their signal peptides and the proximal ends of their secreted sequences align reasonably well, sequence similarity deteriorates rapidly proceeding toward their 3′ ends. We have already identified substantial gene conversion affecting ∼30 bp near the 5′ end of the secreted protein in more than half of the 38 mouse *Esp* genes and nearly all ten of the rat *Esp* genes. This is a significant portion of the relatively small coding regions of many of these genes. Finally, the *Esp* gene expansion appears to be older than that in either the *Mup* or *Abp* genes, possibly predating the divergence of *M. musculus* and *R. norvegicus* [27]. In that case, the *Esp* phylogeny might well be biased by the phenomenon of long branch attraction wherein homoplasy will increase the probability that two lineages will evolve the same nucleotide at the same site [76]. The resulting bias in the gene phylogeny will confound tree-based analyses such as CODEML.

**Figure 4:**
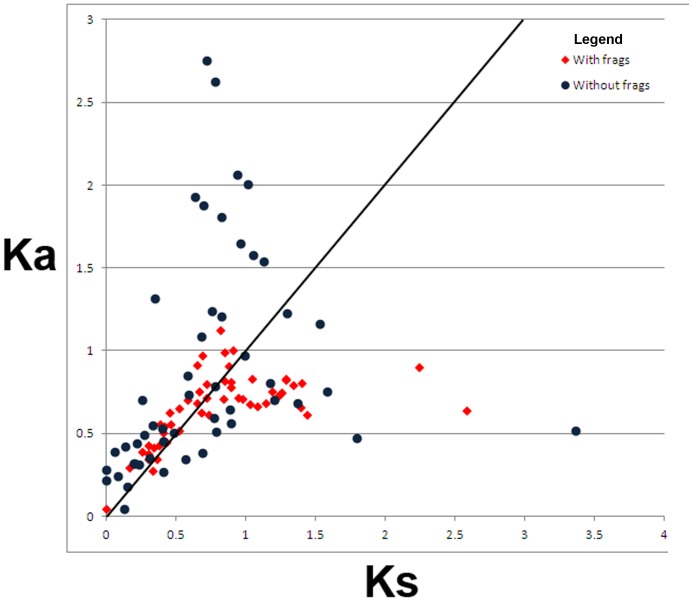
*K_a_* plotted vs. *K_s_* for selected mouse *Esp* sequences. The line demarcates a slope of 1.0. Each sequence is plotted twice. The red diamonds mark the *Esp* sequences including inner fragment sequences and the blue dots show the same *Esp* sequences with the inner fragment sequences removed.

With these caveats in mind, we proceeded with an investigation of the possibility that there has been selection on at least some of the ESPs. Comparison of the signal peptide amino acid sequences encoded in Exon 2 with the secreted protein amino acid sequences in Exon 3, suggests that purifying selection probably has acted on the signal peptides while the secreted portions of the ESPs have undergone much more rapid evolution. Therefore we evaluated the Exon 3 coding regions to obtain rates of nonsynonymous (*K_a_*) and synonymous (*K_s_*) substitutions. As described above, the largest group of inner fragments (25/70) from our GENECONV analysis of *Esp* Exon 3 (*gscale = *1) involve the same *Esp* paralog, *Esp24*, and we chose to focus on those. We sorted that group and retained only those inner fragments that involve *Esp* paralogs shown to be expressed [27]. We further restricted the group to mouse paralogs, reasoning that, although the expansion occurred before the *Mus*-*Rattus* split, selection more recent than that speciation event would have involved paralogs in only one species or the other. That resulted in eleven mouse *Esp* Exons 3 (i.e. from *Esp1, 3, 4, 5, 6, 8, 15, 16, 23, 24, 36*) for analysis. We produced alignments of these, with and without the gene conversion fragment (sites 16–45) and used those for the pairwise *K_a_*/*K_s_* analysis shown in **Fig. 4**.

It is apparent from **Fig. 4** that the majority of data points are grouped near the slope 1 line in the region representing lower *K_a_* and *K_s_* values. Nonetheless, there are numerous values plotted above the slope 1 line, both in the group that contained the gene conversion fragment and in the group from which the fragment had first been removed (28/51, 4 calculations were nullified by DNAsp). Considering the results for the group with the inner fragments removed, we found that all eleven paralogs were present in two or more pairs with *K_a_*/*K_s_* >1.0. *Esp5* appeared in the most pairs (8) and *Esp1* and *Esp24* in the least (2 pairs each, one shared). We note that six *Esp*s (*1, 3, 4, 5, 6* and *8*) of the eleven paralogs listed above are concentrated in one of the major clades of the phylogeny reported by Kimoto *et al* [27] while the other five are distributed among three other major clades. With the caveats stated earlier, we propose that the footprints of positive selection are detectable at least in some mouse *Esp* paralogs.

## Discussion

A number of evolutionary forces may influence the nature of the paralogs that arise from gene duplication, including selection, genetic drift and gene conversion. Purifying (aka negative) selection reduces nucleotide variability among paralogs below a level expected from drift alone, while positive selection promotes nucleotide variability to levels higher than expected from drift. Before attempting to evaluate the extent to which selection contributed to the evolutionary history of a gene expansion, it is important to determine whether concerted evolution influenced the duplication process. Concerted evolution encompasses the processes of ectopic gene conversion and unequal crossing-over that are specific to multi-gene families. The effect of these processes on the expansion of a gene family is that the evolution of the paralogs is not independent [77]–[79], which has significant consequences for interpreting their origins. Gene conversion is considered the primary mechanism of concerted evolution acting on duplicated genes [80], [81] and results when a portion of the DNA sequence of one gene is copied and pasted onto another in the same region of the copied sequence. It is the mechanism we consider here because the effect of the event between duplicated genes, i.e. paralogs, is to reduce the nucleotide variability that may have arisen between them during their divergence, thereby obscuring the effects of selection. An assessment of the contribution of recombination appears in Janoušek *et al* [82].

### What did Gene Conversion Contribute to the Evolutionary Histories of the Pheromone Gene Families?

Several studies suggested that gene conversion played little if any role in the evolution of the *Abp* gene region [13], [22], while a number of other studies documented evidence for significant positive selection in its evolutionary history [42], [45], [64], [83]–[86]. In the case of the *Mup* gene region, Clark *et al* [65] compared the exonic sequences of four mouse *Mup* genes and cDNA sequences and concluded that an ancestral gene conversion event occurred in some exons. More recently, there has been some speculation that gene conversion played a role in the evolution of the *M. musculus* Class B *Mup* genes because of the similarity of the gene coding regions and the proteins they encode [33], [43]. Estimation of *d_N_*/*d_S_* suggested to one of those groups that there was little evidence of positive selection on the *Mup* genes [33]. Here we report the results of the first investigation of the contributions of gene conversion and selection on *Esp* paralogs and we also present data that updates our understanding of the contributions of gene conversion and selection to the *Mup*s.

Our study of gene conversion in the *Mup* genes makes an interesting comparison to the previously documented lack of an appreciable contribution of gene conversion to the mouse *Abp* gene expansion [13], [22] because the GENECONV results we report here suggest that gene conversion has played little if any role in the expansion of the *Mup* gene family. Specifically, we found no evidence for appreciable gene conversion in the *M. musculus* Class A and Class B *Mup* genes and pseudogenes, nor did we find such evidence in the *R. norvegicus Mup* genes. As mentioned in Results (above), GENECONV has low power for detecting conversion events when divergence between duplicates is very low [66] and it has also been shown to have high false negative rates [67]. These limitation would be of greater concern, had we only analyzed the very similar exonic sequences of the mouse Class B *Mup* genes, however, our GENECONV analyses included both the exons and introns of all four *Mup* gene groups we analyzed. This is important because our nucleotide divergences of the Class B *Mup* introns exceed by three-fold those of the exons. Moreover, the collective intron sequence between exons encoding the secreted Class B *Mup*s is 3.6 times as large as the total coding exon size. We conclude that we should have detected more evidence of gene conversion in the *Mup* genes, if it exists, than we did given that gene conversion is not expected to act on exons alone. In summary, even though there are recognized limitations to the GENECONV program, we should have detected a significant level of gene conversion in our analysis of whole *Mup* genes, in spite of the conservation of the coding regions of the Class B *Mup*s. Rather, we argue that the substantially lower nucleotide divergences in the relatively smaller exons most likely reflect the action of purifying selection on the Class B MUPs.

Given this apparently consistent picture of *Mup* and *Abp* gene evolution, it was a striking contrast to find evidence of extensive gene conversion in many *Esp* genes, although we did not find it in all of them. The *Esp* paralogs involved were all found in inner fragments and none in outer fragments. Those in the inner fragments were identified with the same short DNA sequence that ranged from 20–30 bp, depending on whether a mismatch was allowed. Perhaps one of our most important observations was that a number of the *Esp* inner fragments revealed by GENECONV involved both a mouse paralog and a rat paralog consistent with the conclusion of Kimoto *et al* [27] that the *Esp* gene expansion, at least for many/most paralogs, began in an ancestor predating the *Mus*/*Rattus* divergence. There is evidence that the age relationships of the three pheromone gene families are *Esp* (oldest)→*Mup*→*Abp* (youngest) [82]. We conclude that the two youngest gene families, the *Abp*s and the *Mup*s expanded without much contribution from gene conversion, while the expansion of the older *Esp* family shows significant evidence that gene conversion was involved in a region that affected the proximal part of the coding region of the secreted peptides.

### What did Selection Contribute to the Evolutionary History of One or More Pheromone Gene Families?

We applied the CODEML sites analysis to the *Mup* codons as we have done previously for the *Abp* codons [22], [45], [86]. At least two MUP amino acid sites in β-sheets of each of the mouse Class A and Class B MUPs, as well as in the rat MUPs were identified as having evolved under positive selection. These sites are in a β-barrel in the interior of the molecule where they might influence the nature of the ligand preferentially bound. This stands in strong contrast to the ABP sites under selection in both the alpha and beta/gamma subunits, which fall on the surface of one face of the dimer where they could be involved in interaction with other molecules (e.g. receptors; [45], [86]). Nonetheless we cannot rule out that one or more of the MUP surface residues might interact with a receptor(s).

Given the caveats enumerated earlier, we chose to use a different approach to evaluate the possibility that selection has acted on the ESPs, opting to determine *K_a_*/*K_s_* on the Exon 3 sequences with and without the converted sequence segment identified with GENECONV. Our data provide preliminary evidence that at least some *Esp* paralogs experienced positive selection during the expansion of the mouse gene family. Unfortunately, this data does not provide site-specific selection results as was the case with both the ABPs and MUPs, however, it is very likely that CODEML would have given spurious results, particularly as *Esp* alignments deteriorate rapidly proceeding toward their 3′ ends.

### How did Evolution Influence Protein Function?

The products of each of the three gene families seem to have evolved a unique type of function involving some aspect of reproduction. ABPs have been shown to mediate assortative mate selection, based on subspecies recognition that potentially limits gene exchange between subspecies where they meet [21], [23]. In addition, there is evidence that ABP-mediated mate preference across a transect of the European mouse hybrid zone is a case of reproductive character displacement as predicted by reinforcement [87]. Consistent with this, there is now evidence that ABP constitutes a system of incipient reinforcement where *M. m. domesticus* and *M. m. musculus* make secondary contact, the house mouse hybrid zone in Europe [25]. The authors developed and evaluated models for the analysis of the transition of ABP as a trait under reinforcement selection, reporting that the model including a reinforcement parameter showed significantly better fits than a sigmoid cline model.

MUPs have been shown to mediate female recognition of potential mates to avoid inbreeding (for a review, see [29]). MUPs have also been implicated in male–male aggression and have been reported to accelerate puberty in female mice. Several attempts have been made to connect MUP function to subspecies recognition, as has been done with ABP, however, such a connection seems unlikely for several reasons. One reason is that any heritable signal mediating subspecies recognition and discrimination must involve a gene encoding a protein, or a combination of proteins consistently similar among members of each subspecies but significantly different between the two subspecies to be recognizable [24], [25]. The protein itself could be the signal and/or it could be an enzyme producing or a protein binding a subspecies-specific small molecular pheromone that is the signal. In the case of the ABP system, different *Abpa27, Abpbg26* and *Abpbg27* alleles are fixed in *M. m. domesticus* and *M. m. musculus* [86], [88] but that has not been shown to be true of any *Mup* gene [25]. In fact, the signal used in most of the tests suggested to involve MUPs was urine or bedding in which other constituents capable of firing VNO receptors have been identified, in particular sulfated steroids [89], [90] and (methylthio) methanethiol [91]. In short, the specific odorant compounds involved in recognition based on urine have not as yet been characterized [92]. Those caveats aside, the most serious concern stems from the results of actual mate preference tests that show: 1) wild house mice use self-reference matching of MUP patterns to avoid inbreeding [93] and 2) female house mice show a consistent preference for associating with *Mup* heterozygous males over *Mup* homozygous males when heterozygosity across the rest of the mouse genome was controlled [94]. Thus the preponderance of behavioral evidence supports MUP-based disassortative mating, exactly the opposite of the expectations of Vošlajerová Bímová *et al* [25], consistent with the lack of evidence for any *Mup* alleles fixed in different subspecies.

By contrast to the ABPs and MUPs, less is known about the function(s) of the ESPs. At least one of them, ESP1, appears to enhance lordosis and copulation [28], however, the function(s) of the other ESPs are unknown even though at least fourteen of the remaining 37 are expressed [27]. In any event, lordosis is an intrinsic component of copulation and might be expected to have evolved before the recognition functions of the younger two pheromone gene families described above. The *Abp* and *Mup* gene families appear to have expanded relatively recently and rapidly, duplicating numerous paralogs that already had become pseudogenes in the process. This probably occurred by NAHR mediated by LINE1 repeats [82]. On the other hand, the *Esp* gene family expansion appears to be older based on the LINE1 ages calculated by Janoušek *et al* [82]. This is consistent with: 1) the conclusion that the *Esp* gene expansion preceded the mouse-rat divergence (see [27] and our finding that a mouse and a rat paralog sometimes share inner gene conversion fragments); and 2) the evidence that *Abp* [22], [45] and *Mup* [33] gene expansions in the mouse were independent of their expansions in the rat genome.

In the case of ABPs, it should not be surprising that the majority of sites evolving under positive selection are on one face of the surface of the protein [45], [86] and that these are fixed differences between the two subspecies [85], [86], [88]. Those characteristics are expected for a molecule or a combination of molecules consistently similar among members of either subspecies but sufficiently different between the two to be a recognizable signal for subspecies recognition. These subspecies recognition sites likely evolved under cyclical selection of certain amino acid variants [83] that became advantageous at one stage or another in repeated selective sweeps [84], [85]. A recent report suggests that alpha and beta/gamma subunits may have coevolved such sites for harmonious function in the dimeric form that mediates recognition [86].

In the case of MUPs, our data suggest that the role of the bound ligand may have equal or even more importance in recognition than specific sites on the surface of the protein and thus selection might rather be directed at sites on the interior of the β-barrel where ligand binding specificity is determined. This would explain why both classes of mouse MUPs as well as rat MUPs have at least two selected sites on β-sheet secondary structure in the β-barrel. Finding positively selected sites in the mouse Class B *Mup*s is particularly interesting given the conserved sequences in this group [33], [43]. The nucleotide divergence data we report here suggest that purifying selection has had an especially strong role in the evolution of this group compared to the mouse Class A and rat *Mup* genes. Nonetheless the CODEML program was able to ferret out a few specific sites in each group that were subject to positive selection and over half of those were in β-sheet secondary structure in the β-barrel where ligands are bound.

It is relatively easy to envision the need of the ABP and MUP communication systems for evolution of multiple paralogs that play different roles individually, or in combination, to satisfy the need for the kinds of functions described above. This will be especially important if ligands bound by the encoded proteins diversify their functions even more. In both cases, duplication of a progenitor paralog during a rapid and specific gene family expansion, with nucleotide substitutions at nonsynonymous sites driven by positive selection would provide new paralogs with potentially adaptive functions. On the other hand, the need for the number of paralogs in the ESP family is not nearly so clear since the only ESP function known at this time is lordosis mediated by ESP1. While it is tempting to speculate that there are undiscovered functions beyond lordosis that require the number of ESPs that are apparently expressed [27], there is not enough additional information about ESPs to explain the gene conversion among so many mouse and rat paralogs, a biased gene conversion that seems to be under some sort of selection. Nor is there an obvious explanation for the putative positive selection that we have demonstrated here. More work will have to be done on these interesting peptides to shed light on a potential role for diversity in their functions.

## Supporting Information

Figure S1 ***Mup*** **phylogenies used in CODEML analysis. A): Class A** ***Mups*** **; B): Class B** ***Mups*** **.**
(JPG)

Table S1 ***M. musculus*** **and** ***R. norvegicus Mup*** **genes.**
(XLS)

Table S2 ***M. musculus*** **and** ***R. norvegicus Esp*** **genes.**
(XLSX)

Dataset S1 **Putative** ***Rn Esp*** **mRNAs and their predicted proteins.**
(FA) Click here for additional data file.

Dataset S2 **Inner fragments of mouse and rat** ***Esp*** **genes from GENECONV analysis.**
(XLS)

Dataset S3 **Inner fragments of mouse** ***Esp*** **introns b from GENECONV analysis.**
(XLSX)

Dataset S4 **FASTA alignment of** ***Mup*** **sequences encoding secreted proteins.**
(TXT)
