# Supplementary figures and images for: The Roles of Gene Duplication, Gene Conversion and Positive Selection in Rodent Esp and Mup Pheromone Gene Families with Comparison to the Abp Family
Source: PLoS One. 2012 Oct 19;7(10):e47697. doi: 10.1371/journal.pone.0047697 (PMC3477143; doi:10.1371/journal.pone.0047697)

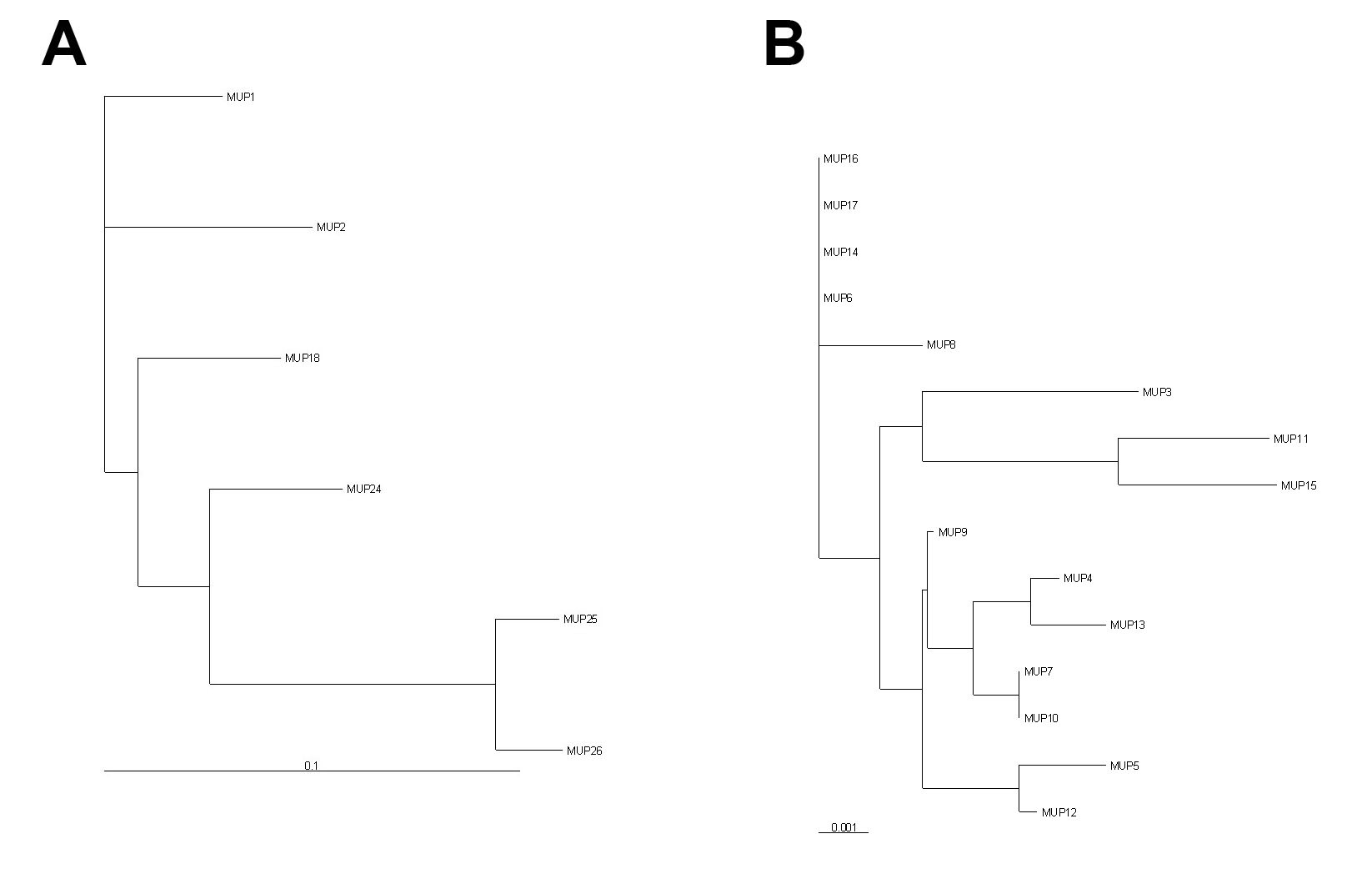

Supplement: Figure S1 — Mup phylogenies used in CODEML analysis. A): Class A Mups ; B): Class B Mups . (JPG) [file pone.0047697.s001.jpg]
